# The Beta-Diversity of *Siganus fuscescens*-Associated Microbial Communities From Different Habitats Increases With Body Weight

**DOI:** 10.3389/fmicb.2020.01562

**Published:** 2020-07-07

**Authors:** Yongjie Wu, Fanshu Xiao, Cheng Wang, Longfei Shu, Xiafei Zheng, Kui Xu, Xiaoli Yu, Keke Zhang, Hongtian Luo, Yufeng Yang, Zhili He, Qingyun Yan

**Affiliations:** ^1^Environmental Microbiomics Research Center, School of Environmental Science and Engineering, Southern Marine Science and Engineering Guangdong Laboratory (Zhuhai), Sun Yat-sen University, Guangzhou, China; ^2^Department of Ecology, Institute of Hydrobiology, Jinan University, Guangzhou, China; ^3^College of Agronomy, Hunan Agricultural University, Changsha, China

**Keywords:** *Siganus fuscescens*, metacommunity, body habitats, body weight, fish-associated microbial community

## Abstract

Fish-associated microbial communities play important roles in host growth, health and disease in the symbiont ecosystem; however, their diversity patterns and underlying mechanisms in different body habitats remain poorly understood. *Siganus fuscescens* is one of the most important consumers of macroalgae and an excellent natural marine source of nutritional lipids for humans, and widely distributes in shallow coastal areas. Here we systematically studied the microbial communities of 108 wild *S. fuscescens* in four body habitats (i.e., skin, gill, stomach, and hindgut) and surrounding water. We found that the β-diversity but not α-diversity of fish-associated microbial communities from each habitat significantly (*p* < 0.05) increased as body weight increased. Also, opportunistic pathogens and probiotics (e.g., *Pseudomongs*, *Methylobacterium*) appeared to be widely distributed in different body habitats, and many digestive bacteria (e.g., *Clostridium*) in the hindgut; the abundances of some core OTUs associated with digestive bacteria, “*Anaerovorax*” (OTU_6 and OTU_46724) and “*Holdemania*” (OTU_33295) in the hindgut increased as body weight increased. Additionally, the quantification of ecological processes indicated that heterogeneous selection was the major process (46–70%) governing the community assembly of fish microbiomes, whereas the undominated process (64%) was found to be more important for the water microbiome. The diversity pattern showed that β-diversity (75%) of the metacommunity overweight the α-diversity (25%), confirming that the niche separation of microbial communities in different habitats and host selection were important to shape the fish-associated microbial community structure. This study enhances our mechanistic understanding of fish-associated microbial communities in different habitats, and has important implications for analyzing host-associated metacommunities.

## Introduction

Fish are continuously exposed to a microbe-rich water environment that circulates through and reaches epithelial barriers of their body. Skin, gill and gastrointestinal tract are the main mucosal surfaces and immune barriers (Gomez et al., 2013), and these mucosal habitats are colonized by complex microbial communities, which interact with the host and regulate host immune systems and nutrient metabolism (Salinas and Magadan, 2017). The balance of these microbial communities is important to the fish development, immunity, health and resistance to external invasions (Fischbach and Segre, 2016; Li et al., 2017; Piazzon et al., 2017). Documenting fish-associated microbial communities can predict their health status (Legrand et al., 2018; Rosado et al., 2019), while determining the extent of variability of fish-associated microbial communities is crucial for healthy aquaculture (Egerton et al., 2018; Martino et al., 2018; de Goffau et al., 2019; Meng et al., 2019). However, a full understanding of fish-associated microbial communities should systematically consider different body habitats (e.g., skin, gill, gastrointestinal tract) with host development, which remains less explored.

Rabbitfish *S. fuscescens*, which widely distributes in tropic and subtropical regions of the western Pacific, belongs to the family Siganidae that only includes the genus of *Siganus* (Oh et al., 2007). The herbivorous *S. fuscescens* mainly consumes algae and seagrasses, and plays an important ecological role in coastal ecosystems. It also facultatively consumes some other plants, detritus, and others if algae and seagrasses are unavailable (Li et al., 2010). As one excellent natural marine source of nutritional lipids, *S. fuscescens* is commercially important marine teleost fish (Li et al., 2010), and it could bio-transform toxic arsenic to detoxify inorganic arsenic (Zhang et al., 2016). In order to improve the economic and environmental benefits of the artificial breeding industry and make reasonable use of various resources, such as seaweeds, various studies have been conducted on fish-associated microbial communities that are closely related to the health of fish. Previous studies with other fish species focused on intestinal microbiota, and indicated that the microbial community composition of the gut was species-specific and influenced by host physiology as well as environmental conditions (Xia et al., 2014; Givens et al., 2015; Guivier et al., 2018). However, little is known about the other body habitats microbial communities, especially in the natural environment.

The architecture and chemical properties of different body habitats may lead to potentially differential niches for shaping the microbial community structure in fish (Guardiola et al., 2014; Chiarello et al., 2015; Friberg et al., 2019). Previous studies showed that mucosal surfaces contained a variety of leukocytes, such as T cells, B cells, plasma cells, macrophages and granulocytes (Salinas et al., 2011), and they provide various niches for microbial colonization and growth. A large difference was observed between internal and external mucosal surface (i.e., gill, skin, and fin) bacterial communities (Lowrey et al., 2015; Guivier et al., 2018; Rosado et al., 2019), suggesting unique and specialized symbiotic relationships at each body habitat. Also, ontogenetic shifts in the fish intestine microbial community composition indicated that fish development substantially affected intestinal microbial communities (Lowrey et al., 2015; Stephens et al., 2016; Li et al., 2017). For example, larval fish tended to have microbial communities for food utilization and from surrounding water (Ingerslev et al., 2014; Stephens et al., 2016; Li et al., 2017), whereas adult fish harbored adaptive and stable microbial communities due to host selection (Llewellyn et al., 2014). More recent evidence showed that microbial diversity differed among body habitats, and it was a strong predictor with many important biological roles even within a single fish (Lowrey et al., 2015; Guivier et al., 2018; Rosado et al., 2019). Although fish intestine and skin microbial communities have been well studied (Burns et al., 2016; Vasemagi et al., 2017; Kashinskaya et al., 2018; Hildonen et al., 2019) at a single stage (Green et al., 2013; Clements et al., 2014), few studies have focused on fish-associated microbial communities from other habitats (e.g., skin, gill) or at different developmental stages.

A metacommunity is a set of communities that are linked by dispersal (Leibold et al., 2004), which considers the scale of interactions beyond the level of individual hosts, thus revealing insights into multiple-habitat environments (Miller et al., 2018). Metacommunity theory has been applied to examine several levels of microbial diversity at different types of habitats (e.g., fish gut, sediment and water), showing that most of metacommunity functional diversity (γ_Ecosystem_) was attributed to local communities (Escalas et al., 2017). Also, the metacommunity analysis from different types of host (e.g., macroalgae, seagrasses) showed that the majority of taxonomic diversity corresponded to inter-habitat differences (Roth-Schulze et al., 2016). The metacommunity theory may advance our understanding of how spatiotemporal dynamics and local interactions shape the community structure and biodiversity (Escalas et al., 2017; Miller et al., 2018). Quantifying the contribution of major ecological processes to the community assembly requires a comprehensive understanding of environmental drivers of microbiome variation across intra-host and inter-host (Costello et al., 2012; Nemergut et al., 2013). Previous studies provided evidences for a mixture of processes in microbial community assembly (Stegen et al., 2012; Burns et al., 2016). For example, Burns et al. (2016) suggested that neutral processes generated a substantial variation in microbial composition across individual hosts although the relative importance of non-neutral processes increased as hosts matured. However, a recent study on gibel carp showed that the gut microbiota was mainly structured by host-associated deterministic processes rather than stochastic processes (Li et al., 2017). However, most such studies were focused on fish intestinal microbial communities, and the microbial communities in other body habitats remains less explored (Lowrey et al., 2015).

In this study, we aimed to understand how the diversity and assembly mechanisms of microbial communities from different body habitats change with fish development in the natural environment. We hypothesized that the overall diversity of fish-associated microbial communities and their potential functions would increase among different habitats as body weight increased due to increased nutrient requirements and immunity for host growth. To test this hypothesis, we investigated *S. fuscescens*-associated microbial communities across four body habitats (e.g., skin, gill, stomach, and hindgut) and compared them with surrounding water microbial communities. We found that niche and host selection could play important roles in shaping the *S. fuscescens*-associated microbial community structure, and they tended to assemble into distinct communities as body weight increased, and the relative abundance of some digestive-related core OTUs increased as body weight increased. This study provides an integrated diversity pattern and assembly mechanisms of *S. fuscescens*-associated microbial communities in different body habitats and their relationships with host body weight, and has important implications for establishing ecologically healthy host-associated metacommunity.

## Materials and Methods

### Site Description and Sampling

Wild *S. fuscescens* were collected from six sites of the Shantou coastal area (Supplementary Figure 1 and Supplementary Table 1) in June, August and October 2017, respectively, and totally we analyzed 108 individuals of *S. fuscescens*. Four tissues of each individual were collected in sterile conditions: (i) 2 m^2^ skin, (ii) 2.0 g gill, (iii) the whole stomach and content, and (iv) 2.0 g hindgut and content. All samples were separately placed into sterile tubes. Also, we took 2.0 L water samples for each sampling sites (both June and August sampled six sites, and only one site in October) and immediately filtered through 0.22 μm filters (Millipore, MA, United States) to collect environmental bacteria and stored at −20°C for DNA extraction as previously described (Yan et al., 2017). All of the samples were stored on ice and shipped back to the lab, then stored at −80°C for subsequent molecular analyses. All protocols involved in the animal experiments were approved by the Institutional Animal Care and Use Committee of the Institute of Hydrobiology, Chinese Academy of Sciences (Approval ID: Keshuizhuan 08529).

### DNA Extraction and 16S rRNA Gene Amplicon Sequencing

We extracted microbial community DNA from the fish tissues and water samples with the following procedures. First, tissues were cut into small pieces and homogenized in 0.1 μM phosphate-buffered saline (PBS) solution using autoclaved mortars and pestles. Second, tissues were rinsed three times with sterile PBS and washing solutions were pooled and centrifuged at 15000 × *g* for 20 min to remove the supernatant and precipitate DNA extraction. MinkaGene Stool DNA kit was then used to extract DNA according to the manufacturer’s instructions. The concentration and quality of extracted DNA were determined using a NanoDrop One spectrophotometer, the purified DNA was stored at −20°C. One skin and five hindgut samples were excluded in subsequent experiments due to the poor quality of DNA, and there were a total of 439 high quality DNA samples (107 skin +108 gill +108 stomach + 103 hindgut+ 13 water samples) available in this study.

A dual-index sequencing strategy was used to amplify the V3-V4 region of the bacterial 16S rRNA gene with universal primers 338F (5′-ACTCCTACGGGAGGCAGCA-3′) and 806R (5′-GGACTACHVGGGTWTCTAAT-3) (Kozich et al., 2013). The amplification conditions were as follows: initial denaturation at 98°C for 2 min, followed by 25 cycles of 98°C for 30 s, 50°C for 30 s and 72°C for 1 min, ending with a final extension at 72°C for 5 min. Library quality was assessed with a Fragment Analyzer, and libraries were subjected to 250 bp paired-end sequencing on a HiSeq platform in Biomarker Technologies Corporation (Beijing, China).

### 16S rRNA Gene Sequencing Data Analysis

Sequence preprocessing was conducted on a publicly available Galaxy pipeline^1^, which integrated all the necessary bioinformatic tools, and parameters of each process were previously described (Kong, 2011; Magoc and Salzberg, 2011; Edgar, 2013). First, sufficiently long (an average fragment length of 253 bp) paired end reads were combined with at least a 30 bp overlap into sequences by FLASH program (Magoc and Salzberg, 2011). Second, the Btrim program, with threshold of Quality Score > 20 and window size > 4, was used to filter out unqualified sequences (Kong, 2011). Any sequences with either an ambiguous base or <200 bp were discarded, and only sequences within the range of 245–260 bp were retained as targeted sequences. UPARSE was then used to remove chimeras and classify those highly quality sequences into operational taxonomy units (OTUs) at an cutoff of 97% identity (Edgar, 2013). An OTU table including 439 samples was normalized with 23,875 reads per sample for downstream analyses.

### Ecological Process Analysis

To estimate the sources of microbial communities observed in different habitats, we used a Bayesian approach for bacterial source-tracking analysis (Comte et al., 2017). Samples from each habitat was designated as sinks, and the rest of samples from other habitats was tagged as sources. In order to directly visualize the role of each habitat for shaping the metacommunity composition, we used an additive diversity-partitioning framework (Belmaker et al., 2008) to decompose the total diversity and expressed it as the sum of the diversity observable at various scales with *Rao* function (Escalas et al., 2013). We decomposed the total diversity into the sum of inter-habitat differences and diversity in local communities with: γ_Ecosystem_ = β_Inter–Habitats_+$\bar{\beta }$_Intra–Habitats_+$\bar{\alpha }$_Local–Community_.

To quantify the influence of ecological processes (e.g., drift, selection and dispersal) on fish-associated and water microbial communities, we calculated the ecological process as previously described (Stegen et al., 2013). In this framework, the variation or turnover of both phylogenetic diversity and taxonomic diversity was first measured with the null model-based phylogenetic and taxonomic β-diversity metrics: β-nearest taxon indices (β-NTI) and Raup-Crick (RC_Bray_). We used β-NTI in combination with RC_Bray_ to quantify the ecological processes that influence *S. fuscescens*-associated and the water microbial community composition on a spatiotemporal scale. If |β-NTI|< 2, the community turnover is governed by heterogeneous or homogeneous selection. Pairwise comparisons with |β-NTI|< 2 were further subjected to RC_Bray_: the fraction of pairwise comparisons with |β-NTI|< 2 and RC_Bray_ < −0.95 estimated the homogenizing dispersal influence; the fraction of pairwise comparisons with |β-NTI|< 2 and RC_Bray_ > 0.95 estimated the dispersal limitation influence; the fraction of pairwise comparisons with |β-NTI| < 2 and |RC_Bray_| < 0.95 represented the component of compositional turnover undominated by any process mentioned above (Stegen et al., 2013).

### Statistical Analysis

To assess the variation in diversity measures among habitats, α-diversity metrics (Chao1 index) were computed, and *t*-test and ANOVA were performed using GraphPad Prism 7.0. For β-diversity, we used principle coordinate analysis (PCoA) to visualize the patterns by the R package. The partial Mantel test was performed between compositional dissimilarity matrices (Bary–Curtis) and a weighted distance matrix with 10,000 permutations in the package vegan (Oksanen et al., 2019). PERMANOVA was used to test the dissimilarity among different habitats, Spearman was used to assess correlations between metadata and feature abundances by GraphPad Prisim 7.0, and liner regressions for multi-feature model building (Guo et al., 2018). To examine the effect of body weight on α-diversity and β-diversity, we used the distance-based linear regression model with an effect of body weight difference.

## Results

### Microbial Diversity Varied Among Body Habitats and Their Relationships With Body Weight

**FIGURE 1:**
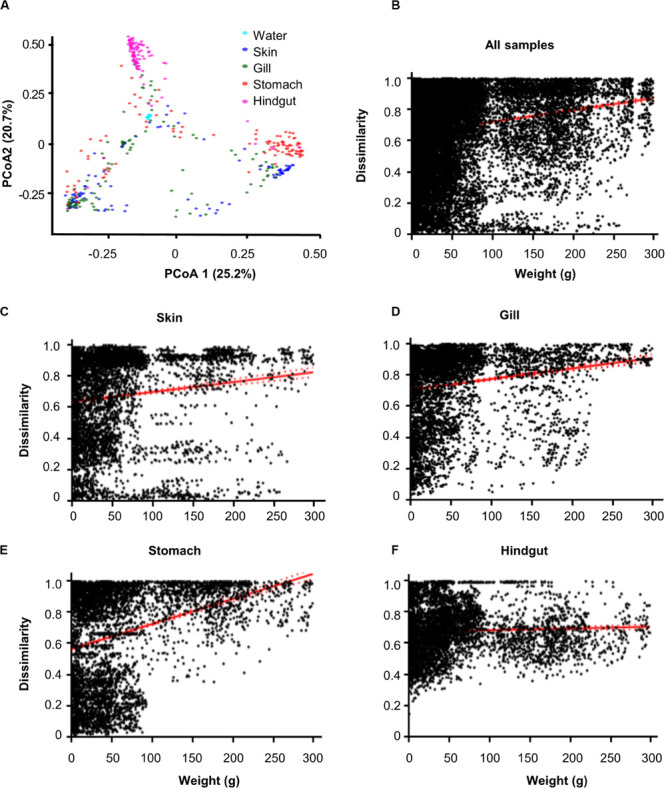
Dissimilarity of microbial communities in different body habitats and its correlations with body weight. **(A)** Principal coordinated analysis (PCoA) based on Bray–Curtis distance. Community dissimilarities significantly (*p* < 0.05) increased as body weigh difference in **(B)** all samples, **(C)** skin, **(D)** gill, **(E)** stomach, and **(F)** hindgut. Solid lines show linear regressions; dotted lines show 95% confidence interval of regression lines. The *p*-value was calculated by comparing the observed *F*-value with those from 1,000 randomized data sets.

The 16S rRNA gene amplicon sequencing obtained 13,543, 14,290, 14,420, 8,052, and 5,650 operational taxonomic units (OTUs, 97% cutoff) from habitats of water, gill, skin, stomach and hindgut, respectively (Supplementary Figure 1 and Supplementary Table 1). The results showed that α-diversity (Chao1) was significantly (*p* < 0.01) different among habitats (Supplementary Figure 2A). Specifically, the water microbial community exhibited the highest α-diversity, followed by the hindgut, gill, stomach, and skin microbial communities. However, Spearman’s correlation analyses did not show significant (*p* > 0.05) correlations between the α-diversity and fish body weight (Supplementary Figures 2B–F). Principle coordinate analysis (PCoA) showed significant (*R* = 0.1427) differences among five habitats, while hindgut and water habitats were closely clustered (Figure 1A). Furthermore, we found significant overall relationships between β-diversity and body weight for each individual habitat or for all habitats (slope = 0.0001–0.0023, *p* < 0.001) (Figures 1B–F). It is noted that the hindgut microbial communities only showed a weak correlation as body weight increased (slope = 0.0001).

### Microbial Compositions Varied Among Habitats and Changed as Body Weight Increased

From 439 samples investigated, we detected 25 phyla and 411 genera. The five most abundant phyla accounted for >95% of detected sequences, and their abundances varied in different habitats (Supplementary Figure 3 and Supplementary Table 2). At the phylum level, the relative abundance of Firmicutes in gill, Cyanobacteria and Actinobacteria in skin significantly (*p* < 0.05) decreased with body weight. However, Proteobacteria in skin significantly (*p* < 0.05) increased as weight increased (Supplementary Figure 4). At the genus level, the variation of microbial composition was far more different among body habitats than that at the phylum level (Supplementary Table 3). Specifically, *Pseudomonas*, *Synechococcus*, *Methylobacterium*, *Streptococcus*, and *Ralstonia* were abundant and widely distributed in *S. fuscescens* body habitats. The relative abundance of *Pseudomonas* in skin, *Streptococcus* in stomach significantly (*p* < 0.05) increased with body weight. However, *Methylobacterium* and *Synechococcus* in skin, *Streptococcus* in gill significantly (*p* < 0.05) decreased as body weight increased (Supplementary Figure 5).

### Core Microbial Taxa Among or Within Habitats

**FIGURE 2:**
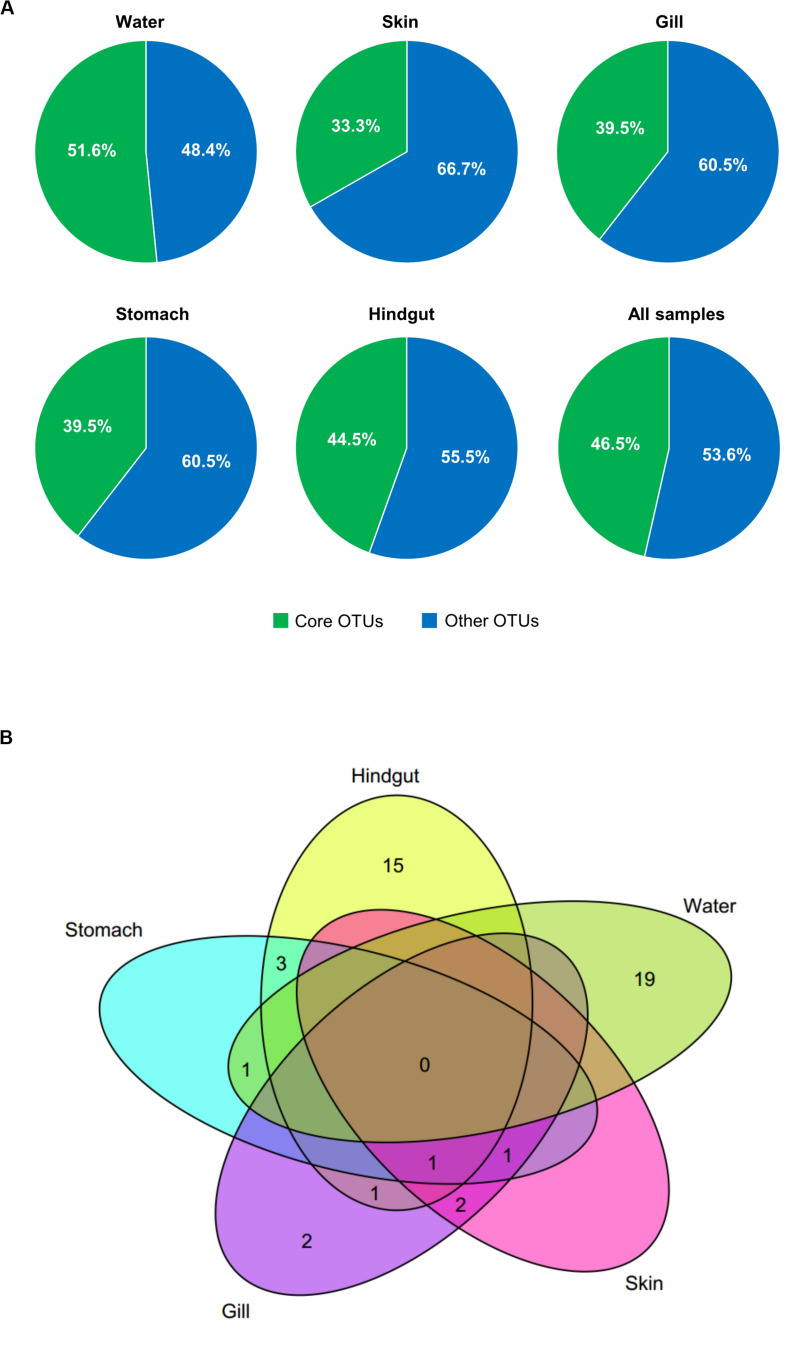
Core OTUs (shared by more than 80% samples and relative abundance >1%) at different habitats. The relative abundances **(A)** and Venn diagram showing the number of shared and unique core OTUs **(B)** in different habitats.

**FIGURE 3:**
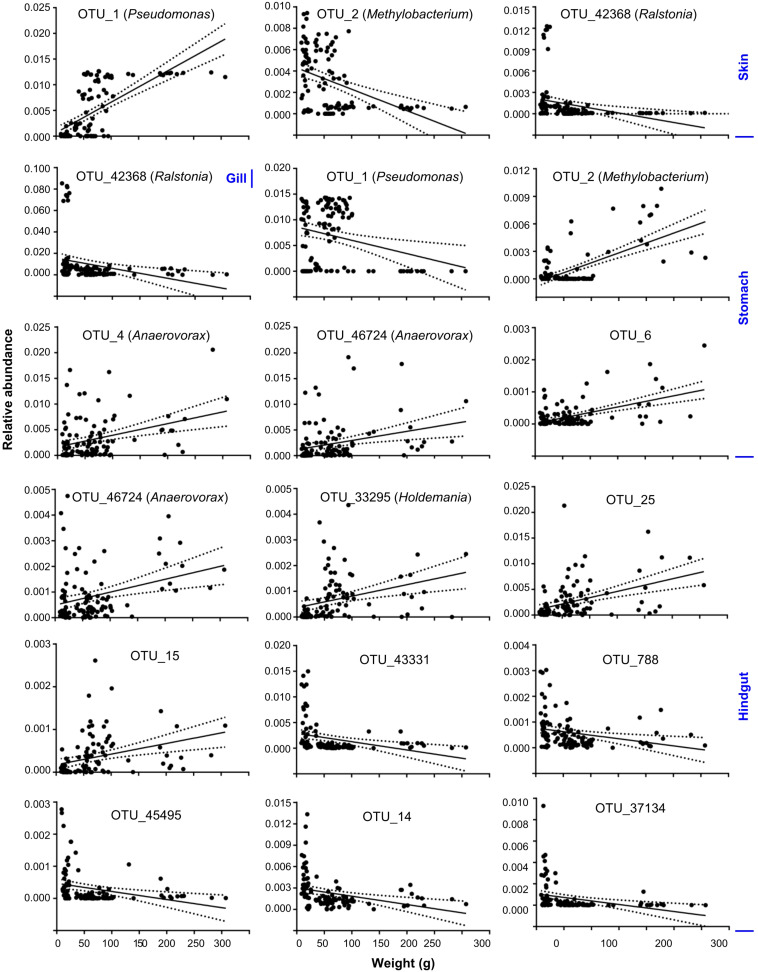
Core OTUs significantly changed as body weight increased at skin, gill, stomach and hindgut. Solid lines show linear regressions; dotted lines show 95% confidence interval of regression lines.

To better understand the variation of microbial communities among body habitats, “core” OTUs were identified within each habitat. We defined that core OTUs were detected in more than 80% samples, and with abundance >1% for each sample of the considered habitats. Only a *Pseudomonas* OTU (OTU_1) was identified as a core taxon in all body habitats, reflecting its universality in *S. fuscescens*. For each habitat, the water, skin, gill, stomach and hindgut microbiomes had 20, 4, 7, 6, and 20 core OTUs, respectively (Figure 2A and Supplementary Table 4), which correspondingly accounted for 48.4, 60.5, 66.7, 60.5, and 51.5% of the total abundances in each habitat, respectively (Figure 2B and Supplementary Table 4). OTU_2 was closely related to *Methylobacterium*, and highly abundant in gill, skin and stomach. Especially, three core taxa associated with digestive bacteria, “*Anaerovorax*” OTUs (OTU_6 and OTU_46724) and “*Holdemania*” OTU (OTU_33295) were identified as core OTUs in the hindgut and their abundances increased as body weight increased (Figure 3). However, there was no core OTU between the four fish body habitats and water environment.

### Contributions of Microbial Communities From Different Habitat to the Metacommunity Assembly

**FIGURE 4:**
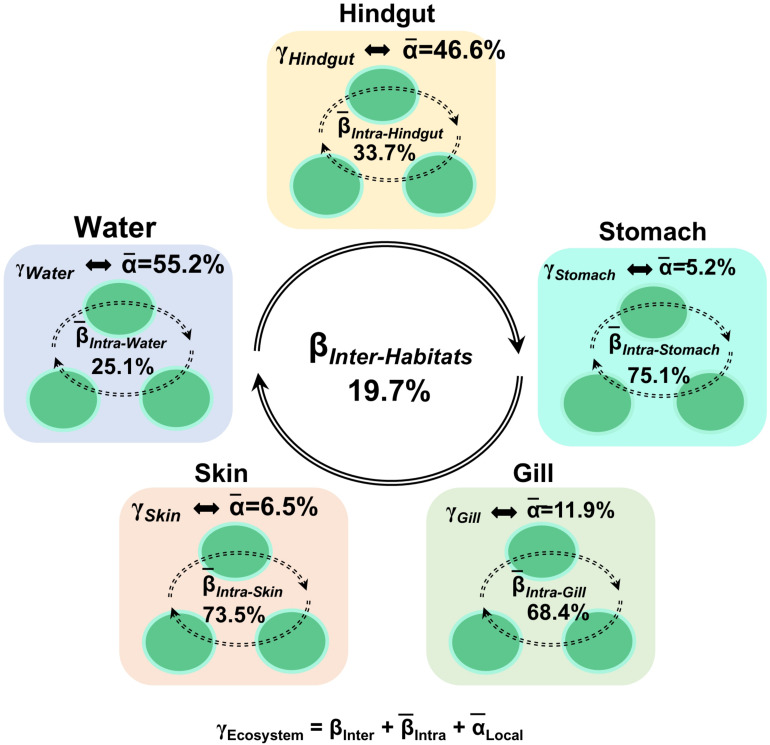
Multi-scales hierarchical partitioning of variant diversity. The total diversity at the ecosystem level (γ_Ecosystem_) was partitioned into the contribution of skin, gill, stomach, hindgut and water. We expressed this total compositional diversity within the ecosystem as the sum of inter-habitat compositional difference (β_Inte__r__–Habitats_), the mean intra-habitat compositional difference (¯β_Intra–Habitats_) and the mean local diversity (¯α_Local–Habitats_) by γ_Ecosystem_ = β_Inter–Habitats_ +(¯β_Intra–Habitats_) +(¯α_Local–Habitats_).

**FIGURE 5:**
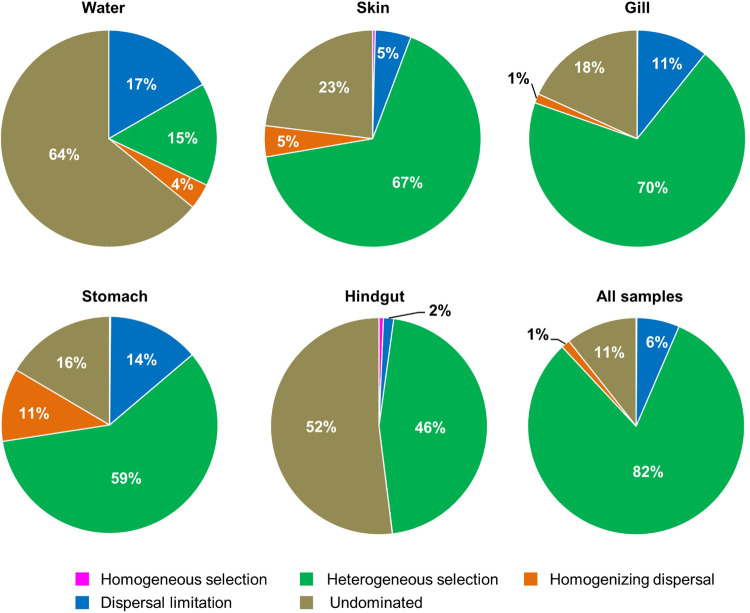
The contribution of ecological processes that governing the assembly of fish − associated microbial communities and water microbiotas among habitats.

To understand the relative importance of each habitat toward the metacommunity, Source-Tracker analysis was performed to evaluate the proportion of different dispersal sources for the community composition of each habitat. Our results indicated that the hindgut microbial community was particularly sourced from the stomach (83%) (Supplementary Figure 6). However, microbial communities of different body habitats were not mainly sourced from water (6–11%). The holistic insights of the diversity contribution to the metacommunity indicated that $\bar{\beta }$_Intra–Habtats_ contributed 55.2% of the total diversity (γ_Ecosystem_), outweighing the contribution of $\bar{\beta }$_Inter–Habitats_ (19.7%) and $\bar{\alpha }$_Local–Community_ (25.2%) (Figure 4). Specifically, $\bar{\beta }$_Intra–Hindgut_ (33.7%) and $\bar{\beta }$_Intra–Water_ (25.1%) were lower than $\bar{\beta }$_Intra–Gill_ (68.4%), $\bar{\beta }$_Intra–Skin_ (73.5%) and $\bar{\beta }$_Intra–Stomach_ (75.1%), indicating that microbial dispersal in the gill, skin and stomach may be limited. This relatively high contribution of β-diversity to γ_Ecosystem_ revealed the importance of inter-habitats communities for ecosystem diversity. To further gain insights into microbial community assembly mechanisms, we quantified the ecological processes governing the fish-associated microbial communities and water microbial communities. In the hindgut, the proportion of deterministic selection (47%) and undominated (52%) processes were similar, while deterministic processes were the main forces for microbial assembly in the gill (70%), skin (67%) and stomach (59%) (Figure 5). Conversely, undominated (64%) and stochastic dispersal (21%) played a key role in shaping the water microbial community structure.

## Discussion

Microbial communities in different fish body habitats and surrounding water environments play important roles in fish growth, development and health by providing nutrients, promoting immunity, and defensing pathogen infections. In this study, we found that the β-diversity of fish-associated microbial communities from all tested habitats increased as body weight increased, and the heterogeneous selection appeared to be the major force for the metacommunity assembly. Also, the fish-associated metacommunity was assembled into distinct microbial communities as body weight increased, which was largely driven by niche and host selection. These results generally support our hypothesis that the overall diversity of fish-associated microbial communities would increase with the host development.

Host development may make major shifts of microbial community composition, dominant microbes, and core taxa (Stephens et al., 2016). Previous studies showed that intestinal microbial diversity increased through gibel carp development (Li et al., 2017), while it decreased as zebrafish developed, and finally remained relatively stable in the adulthood (Stephens et al., 2016). This discrepancy may be due to the fact that the fish-associated microbial communities could be affected by diet, environmental and genetic conditions (Lowrey et al., 2015; Guivier et al., 2018; Friberg et al., 2019) as well as the continuous in-flux of contents along the digestive system (Konopka et al., 2015). However, most previous studies were focused on the intestine, while other body habitats were poorly understood. In this study, we showed that the α-diversity of *S. fuscescens*-associated microbial communities was not correlated with body weight, but the β-diversity increased as body weight increased in all body habitats tested, suggesting that the dissimilarity of *S. fuscescens*-associated microbial communities increased as body weight increased. This finding is consistent with previous studies of microbial communities across fish development (Stephens et al., 2016; Yan et al., 2016), showing the intestinal microbial community varies across host development.

It is widely accepted that growth and development would increase nutritional demands, and a change of gut microbial composition may directly degrade complex components (e.g., cellulose) into absorbable nutrients (Turnbaugh et al., 2006) or may allow hosts to digest foods more efficiently to meet their energy and nutrient requirements (Amato et al., 2014). Previous studies have found that some herbivores (e.g., grass carp) are unlikely to have enzymes for degrading major components (e.g., cellulose, hemicellulose, lignin) of plant diet, and require an aid of symbiotic microbiomes to digest them and make energy and nutrients available to the host (Clements et al., 2009). *Clostridium*, *Anoxybacillus*, and *Leuconostoc* genera are known as cellulose-degrading bacteria and were detected from the gastrointestinal microbiota, showing that they are important members of anaerobic cellulose degradation, enabling fish to utilize fibrous plant materials efficiently as nutrients (Wu et al., 2012; Hu et al., 2014). In this study, *Clostridium* were abundant in the hindgut but showed no correlation with body weight. These results appear not to fully support our hypothesis with three possible reasons. First, *S. fuscescens* is facultative omnivorous. Although it generally uses plant materials (e.g., macroalgae), it also eats some animal carcasses (Li et al., 2010). Second, within the sampled body weight range, the sampled fish were already at or near adulthood, and their intestinal microbiome of fish had been succeeded to a relatively stable community. Third, the adult fish selectively feeds on relatively stable foods, and may need particular microbes’ help to digest foods (Stephens et al., 2016; Yan et al., 2016). This is consistent with previous studies that fish-associated microbial communities at the early development were more sensitive to diets and environments than those at the adulthood (Austin, 2006), and remains relatively stable in adulthood (Stephens et al., 2016; Yan et al., 2016). Finally, intestinal microbial communities of wild fish in this study were greatly affected by various factors such as environment and host, so it is expected to further examine such issues under laboratory rearing conditions in the future. Therefore, our results indicate that plant biomass degrading microbes were abundant but they were not affected by body weight.

Different body habitat architecture and chemical properties of the secreting substances can lead to differences in potential niches for shaping the microbial community structure (Salinas et al., 2011; Salinas, 2015), and microbial species specific to different mucosal sites may perform essential physiological or metabolic roles. Previous studies showed that body habitat was a strong determinant factor for the composition of microbiomes, and the microbial communities were distinct between external and internal body habitats (Lowrey et al., 2015; Guivier et al., 2018; Friberg et al., 2019). Higher α-diversity was observed in rainbow trout external body habitats (skin, gill and olfactory organ), whereas the gut could offer more stable habitats that shape specialized microbial communities (Lowrey et al., 2015). Two factors may contribute to these findings. The different sources of microbes in contact with mucosal surfaces probably account for the major differences in the microbiota between external body habitats (e.g., the skin, caudal fin and gills, in contact with the surrounding water), and internal body habitats (e.g., the gut, in contact with microbes in diet). The physiology of the local host body habitat, and their immune responses in particular, may select for a microbiota with a specific composition (Salinas, 2015; Kelly and Salinas, 2017). Also, the composition of mucus, pH, temperature, and oxygenation can also have selective effects on microbiota. These results suggested a reflection of niche, environmental diversity and genetic filters on shaping the microbial community structure, implying the deterministic assembly of microbial communities in different habitats. However, we observed a higher α-diversity in the hindgut than that in other body habitats, and the stomach was not different from the external body habitats (skin and gill). It may be because microbial communities on the skin and gill surfaces are entirely associated with mucus layers swept by water currents (Nava et al., 2011), whereas the hindgut contains microbiota within mucus layers and the content of water currents, which provides a massive source of substrates (Leung et al., 2018). Further research on how body habitats affect fish-associated microbial communities, and what their specificity is defined for each body habitat is needed.

Gill, skin and intestine are the main mucosa-associated lymphoid tissues of fish, and harbor diverse pathogenic and antimicrobial bacteria, which contribute to host health and disease (Llewellyn et al., 2014). For example, *Pseudomonas, Methylobacterium, Vibrio, and Photobacterium* were found abundant in fish with both pathogenic and probiotic species (Benhamed et al., 2014; Banerjee and Ray, 2016). Previous studies showed that *Pseudomonas* species could inhibit the growth of *Saprolegnia australis* and *Mucor hiemalis in vitro* dual culture assays (Lowrey et al., 2015), and they also contain potential secondary bacterial pathogens in fish with high lice infestation (Llewellyn et al., 2014). *Methylobacterium* species are associated with probiotic and/or anti-microbial activity and inhibit the growth of pathogens by biosynthesis of antibiotics (Llewellyn et al., 2014), which is known to be important to host health (Kovaleva et al., 2014). For example, *Methylobacterium rhodesianum* could produce poly-β-hydroxybutyrate to inhibit the growth of pathogens like enterobacteria and *Vibrio* sp. (Boutin et al., 2013). *Clostridium* species, such as *Clostridium butyricum* have been used successfully as a probiotic to stimulate the immune responses and improve survival of Japanese flounder *Paralichthys olivaceus* (Taoka et al., 2006). However, other *Clostridium* species, like *Clostridium difficile* are associated with diarrheal disease in humans and animals (Metcalf et al., 2011), but such a phenomenon was not observed in the fish microbiome. *Vibrio* species worked well to protect against *Aeromonas salmonicida* and *Vibrio ordalii* as a probiotic for *Atlantic salmon in vivo* (Austin et al., 1995); however, they could also infect larvae and cause sudden emergency (Chen et al., 2015). *Photobacterium* species were commonly found on the surface of healthy fish; however, some *Photobacterium* could produce harmful enzymes. For example, *Photobacterium damselae* is a neuraminidases producer and associated with skin ulcers, and it is also an infectious agent of *pasteurellosis* in fish (Urbanczyk et al., 2011). These studies indicate that pathogenic microorganisms are integral components of fish microbiomes, and their presence may not often cause diseases (de Bruijn et al., 2018). In this study, we found that all these bacteria were relatively abundant in *S. fuscescens* body habitats. *Pseudomonas* and *Methylobacterium* were abundant in skin and gill, suggesting that gill and skin are important immune protective barriers against foreign invasions. Our results support the ideas that microbial consortia rather than single microbial species determine host health and disease (Gilbert et al., 2016). However, both *Pseudomonas* and *Methylobacterium* were remained stable at the sampled weight range, which may be due to the fact that the fish immune system had been matured (generally a few weeks after birth) at the sampled weight range (Burns et al., 2017).

Characterizing the composition and structure of microbial communities is the basis for understanding their ecological roles and assembly mechanisms (Trosvik and de Muinck, 2015). It is especially necessary to take a multi-scale study to reveal how local and regional factors affect the community assembly processes that drive emergent patterns. However, most studies of healthy individuals were focused on a single body habitat, particularly in the gut or skin (Martinson et al., 2017; Chen et al., 2018; Sherrill-Mix et al., 2018). A previous study on skin-associated communities (dorsal, anal, pectoral, and caudal fins) of two teleost fish showed that the inter-individual and intra-individual dissimilarities were particularly high (Chiarello et al., 2015), indicating that environmental filtering and body physical isolation play an important role in the assembly of these communities (Costello et al., 2009; Lowrey et al., 2015). A recent study also showed that the gut microbiome was mainly shaped by the gut environment and by some other selective changes accompanying the host development process, suggesting that stochasticity increased and determinism decreased in the adults (Yan et al., 2016). In this study, we found that the contribution of β-diversity to γ_Ecosystem_ was greatly outweighing that of $\bar{\alpha }$_Local–Community_, indicating large differences among habitats, and this may be largely due to the differences in local factors (e. g., the components of mucus surfaces in different habitats, pH), or from regional factors (e.g., limitation in dispersal rates between them) (Martinya et al., 2011). Also, we found that the structure of *S. fuscescens*-associated microbial communities was mainly governed by heterogeneous selection, while undominated forces were important in shaping the water community structure, indicating that dispersal was limited among *S. fuscescens* body habitats. This could be due to physiochemical environments, niche availability and microbial interactions (Costello et al., 2009; Lowrey et al., 2015). Both diversity patterns and ecological processes suggested that habitat separation could alter the microbial communities by reducing their dispersal. These results support the concept that the metacommunity can provide important insights in contrast with that conventionally restricted to local communities alone (Miller et al., 2018). Our results are generally consistent with previous studies, showing that dispersal could reinforce homogenization of local communities, which constitute a major process through which diversity accumulates in local microbial communities by decreasing β-diversity and increasing α-diversity (Cadotte, 2006; Chase et al., 2011).

## Conclusion

We found that the β-diversity but not α-diversity of fish-associated microbial communities from each habitat increased as body weight increased. This study provides new evidence for ecological configurations of microbial communities across fish body habitats. *S*. *fuscescens*-associated microbial communities varied among different body habitats, and tended to assemble into distinct communities as body weight increased. Also, we found some abundant core taxa and genera related to digestion increased as body weight increased. Considering *S. fuscescens*-associated microbial communities from different body habitats as a metacommunity greatly enhances our understanding of ecological mechanisms in the natural environment during host development.

## Data Availability Statement

The raw sequencing data can be found at the EMBL (https://www.ebi.ac.uk/ena) under an accession number (PRJEB37803).

## Ethics Statement

The animal study was reviewed and approved by the Institutional Animal Care and Use Committee of the Institute of Hydrobiology, Chinese Academy of Sciences (Approval ID: Keshuizhuan 08529).

## Author Contributions

QY, ZH, CW, and LS conceptualized and supervised the study. FX, CW, and KX performed the data curation. YW, XZ, and KZ contributed to the formal analysis. QY and ZH contributed to the funding acquisition and project administration. YW, FX, XZ, KX, XY, and KZ investigated the study. YY and HL performed the resources. YW, FX, XZ, and KZ visualized the manuscript. YW and FX wrote the original draft of the manuscript. All authors contributed to the data analysis and interpretation and commented on the manuscript.

## Conflict of Interest

The authors declare that the research was conducted in the absence of any commercial or financial relationships that could be construed as a potential conflict of interest.

## Footnotes

1 http://mem.rcees.ac.cn:8080
