# Supplementary material for: The Beta-Diversity of Siganus fuscescens-Associated Microbial Communities From Different Habitats Increases With Body Weight
Source: Front Microbiol. 2020 Jul 7;11:1562. doi: 10.3389/fmicb.2020.01562 (PMC7358552; doi:10.3389/fmicb.2020.01562)
Supplement: Supplementary file 2 [file Data_Sheet_1.docx]

**Supplementary Figures**

**
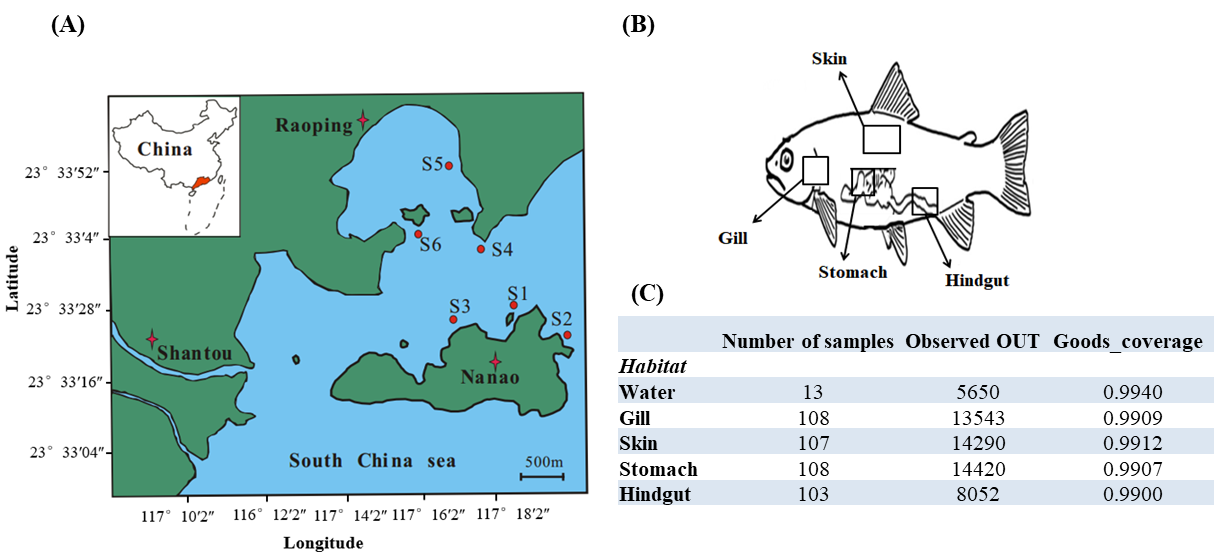
**

**Supplementary Figure 1.** The overview of *S. fuscescens* samples. (A) Sampling sites in Shantou Nanao Isand and Raoping. 108 wild *S. fuscescens* with different sizes (weight 8.9 ~307.4 g) were captured from six coastal sites in June, August and October 2017, respectively. The distance between any two of these sites is about 600-3,000 meters. (B) Diagram of the tissue sampling strategy for the present study. (C) Overview of *S. fuscescens* samples including groups, quality sequences and core OTU number (abundance > 1%). Operational taxonomic units (OTUs) are defined at 97% sequence similarity.


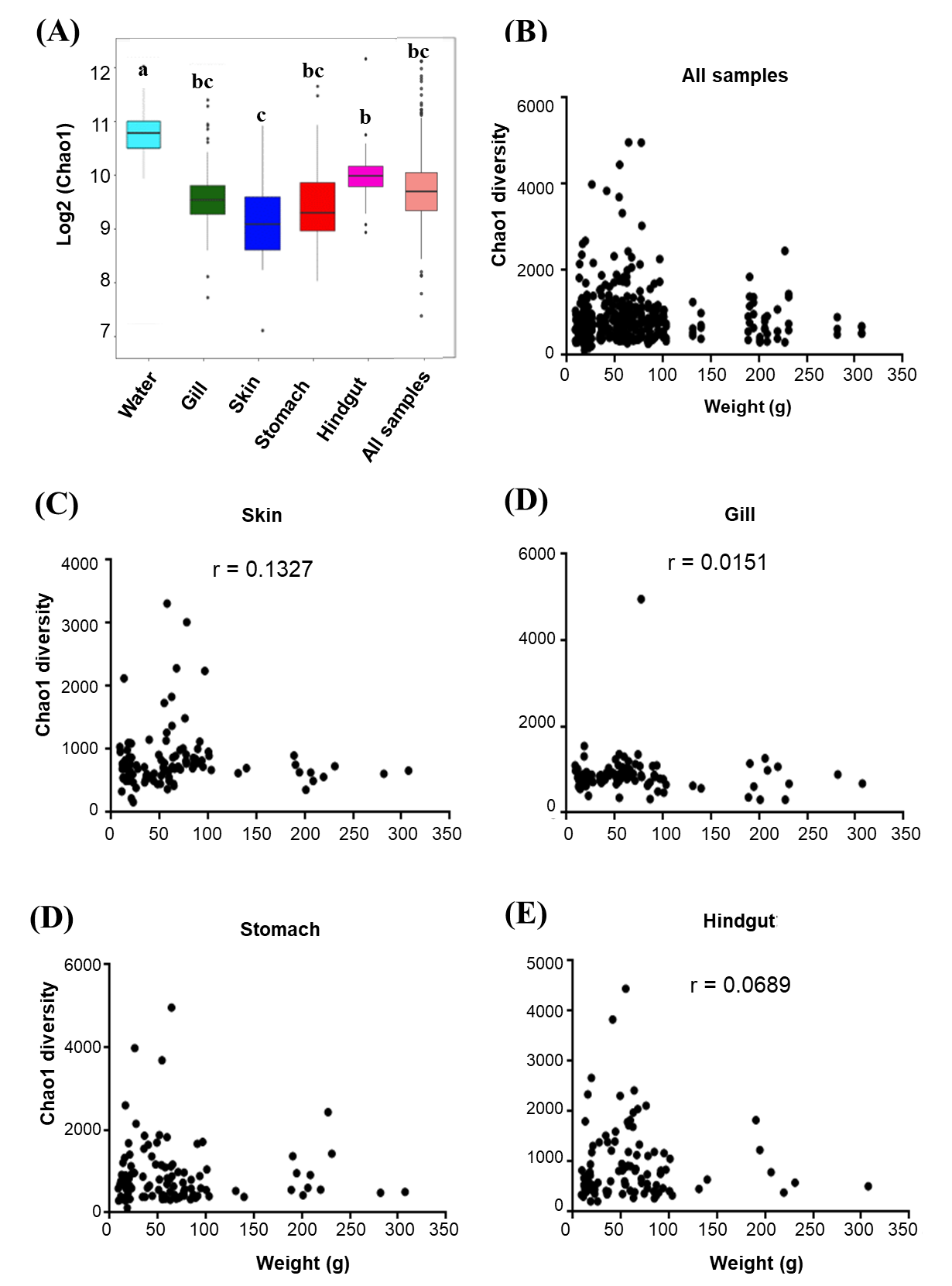


**Supplementary Figure 2.** Bacterial biodiversity of *S. fuscescens* at different body habitats and correlation with body weight. (A) The Chao1 diversity of microbial communities from different body habitats of *S. fuscescens*. ANOVA multiple comparisons were used to test the significance among body habitats. The relationship between body weight and Chao1 diversity in (B) all samples, (C) gill, (D) skin, (E) stomach and (F) hindgut.


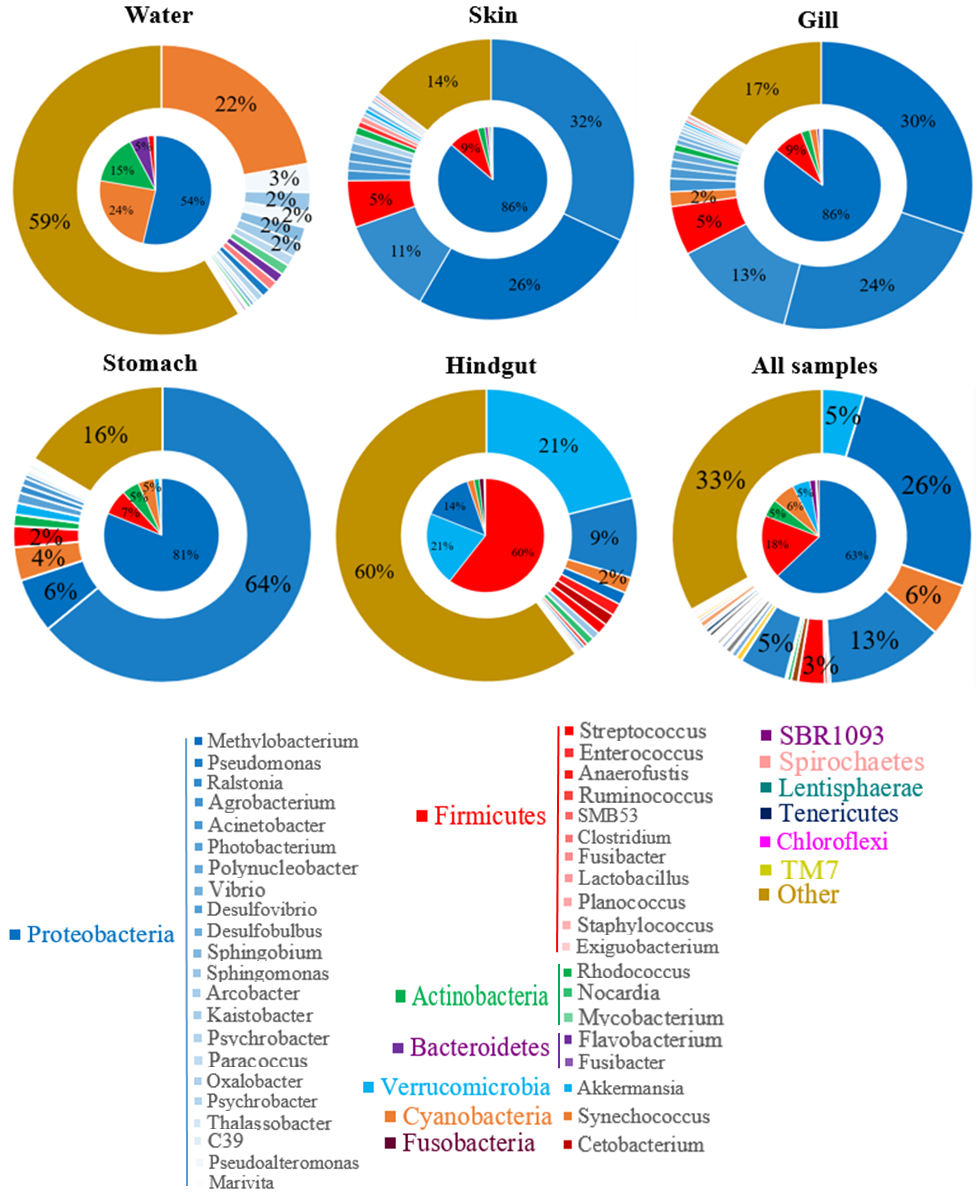


**Supplementary Figure 3.** The microbiome of various anatomical locations of the fish body. The relative abundance of most abundance phyla and genera in five habitats with the inner ring at the phylum level, the outer ring at the genus level, and the gradient of the same color belong to different genera within a phylum.


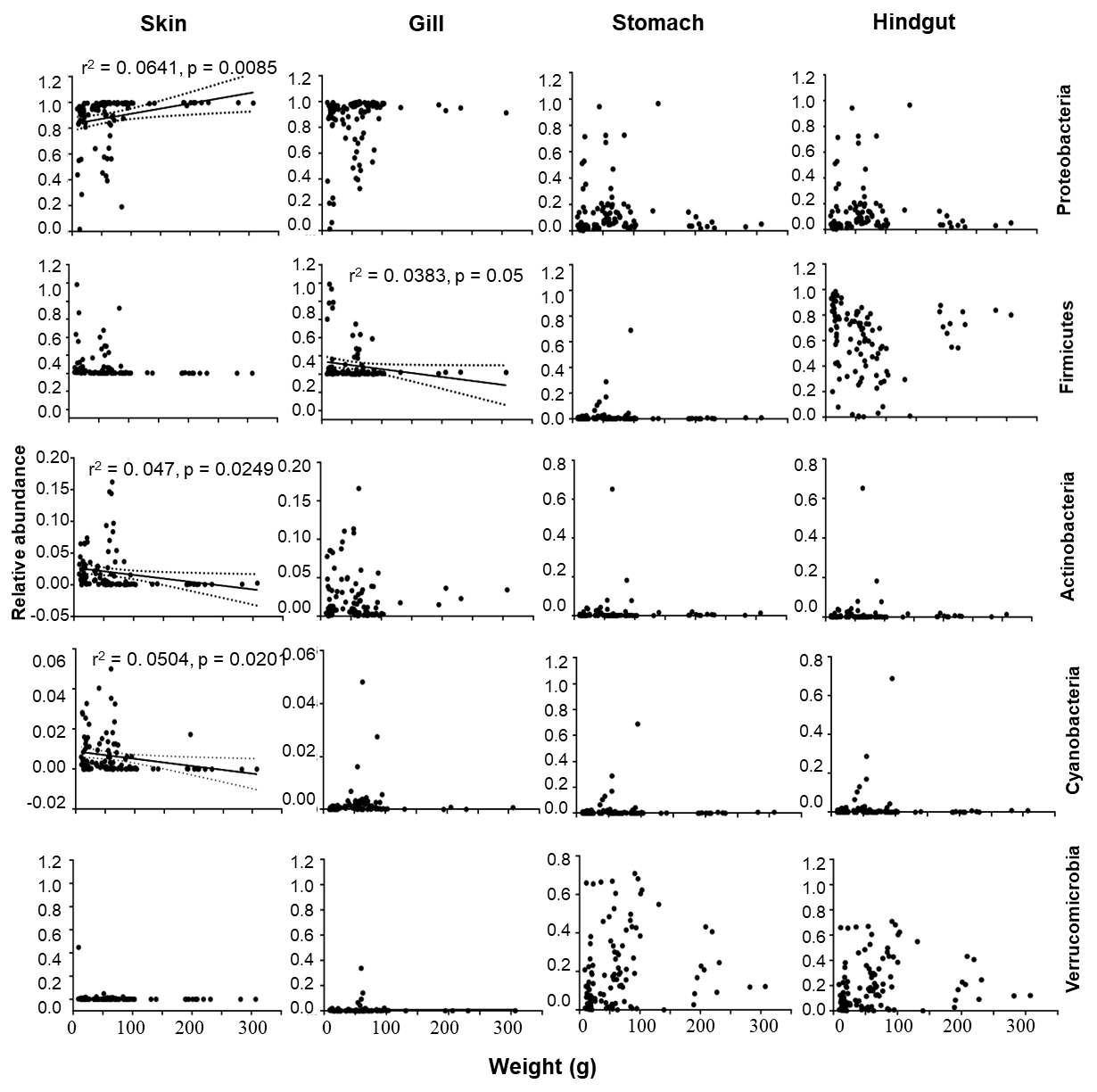


**Supplementary Figure 4.** The change of most abundance phyla: Firmicutes, Proteobacteria, Verrucomicrobia, Cyanobacteria and Actinobacteria in different body habitats as body weight increased at skin, gill, stomach and hindgut with 95% confidence interval (CI) of regression lines in different body habitats at genus level.


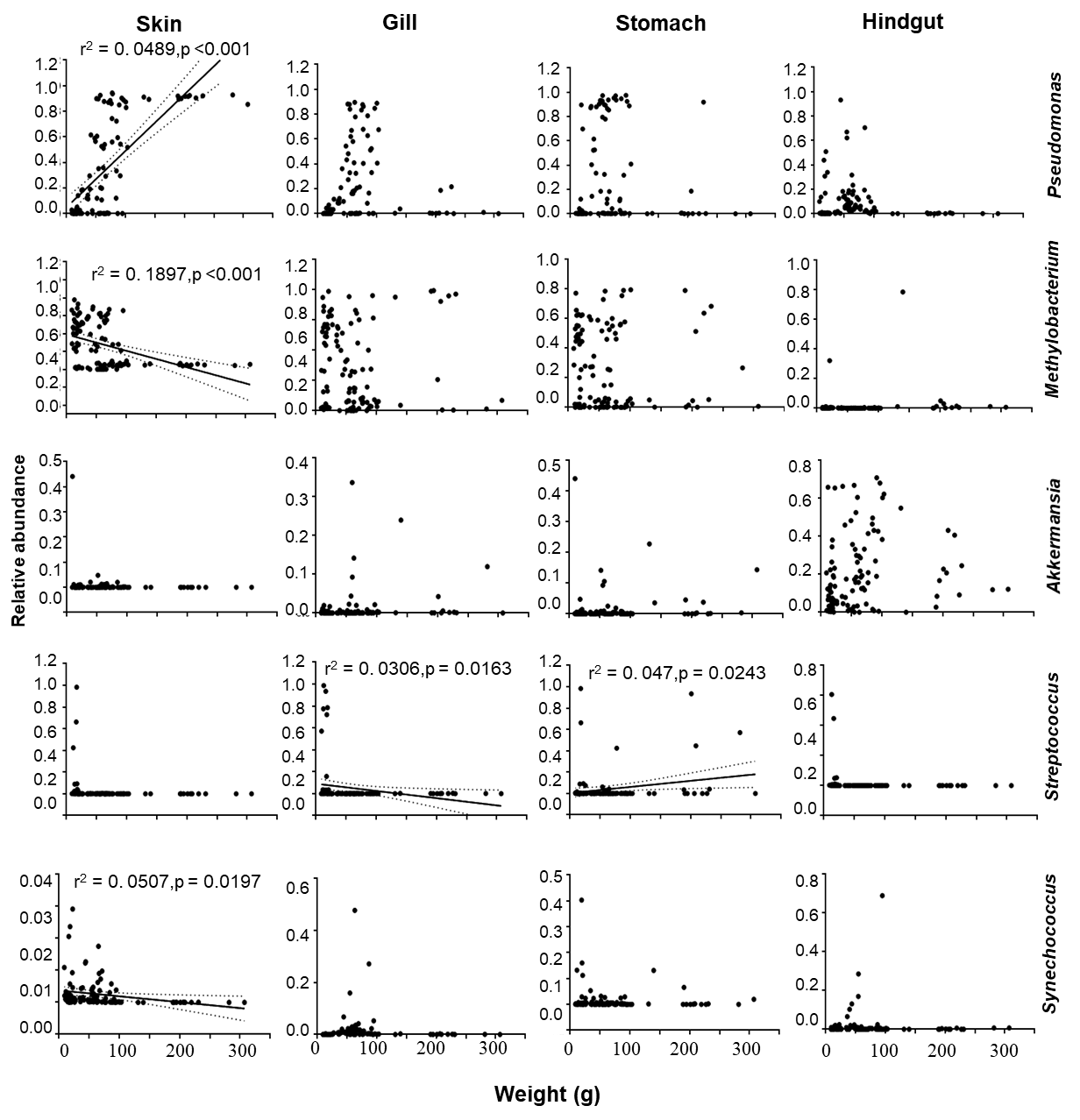


**Supplementary Figure 5.** The change of most abundance genus: *Pesudomonas*, *Methylobacterium*, *Akkermansia*, *Streptococcus* and *Synechococcus* in different body habitats as body weight increased at skin, gill, stomach and hindgut with 95% confidence interval (CI) of regression lines in different body habitats at genus level.


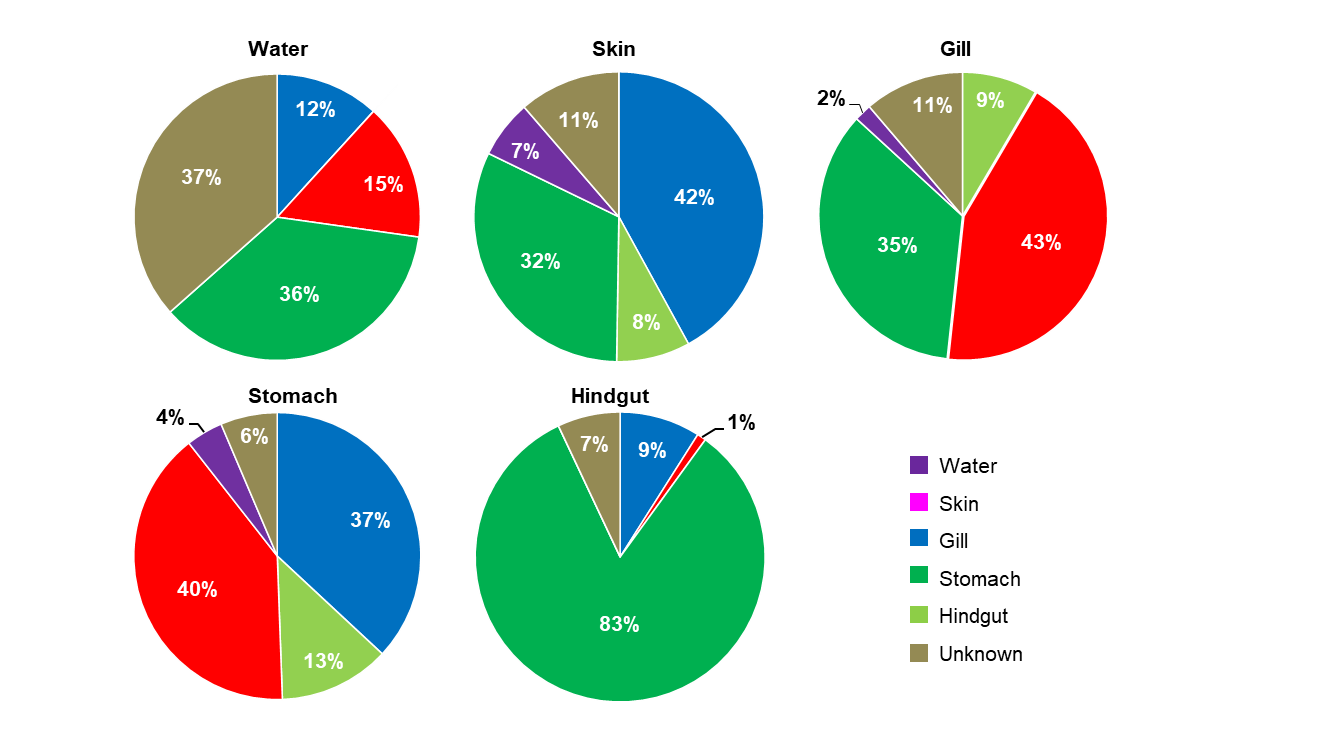


**Supplementary Figure 6.** Source-Tracker analysis of source contributions to the sink. The contribution of different source communities to each habitat, respectively.

**Supplementary tables**

**Supplementary Table 1.** Overview of *S. fuscescens* samples including groups, sampling time, sampling body habitat, body weight, Shannon diversity, Chao1 diversity, Observer_otu and Goods coverage, and see in the XLS file.

**Supplementary Table 2.** The most abundant bacterial groups at phyla level were detected in different habitats (gill, skin, stomach, hindgut and water). ANOVA multiple comparisons were used to test the significance among habitats.

|  | **Water (%)** | **Gill (%)** | **Skin（%）** | **Stomach (%)** | **Hindgut (%)** | **All samples (%)** |
| --- | --- | --- | --- | --- | --- | --- |
| **Proteobacteria** | 53.95^c^ | 81.82^ab^ | 88.28^a^ | 78.10^b^ | 13.71^d^ | 62.90^c^ |
| **Firmicutes** | 2.11^b^ | 11.13^b^ | 6.98^b^ | 10.17^b^ | 60.33^a^ | 18.07^b^ |
| **Actinobacteria** | 14.26^a^ | 2.51^c^ | 1.98^c^ | 5.00^b^ | 1.42c | 5.01^b^ |
| **Cyanobacteria** | 23.52^a^ | 1.90^bc^ | 0.66^c^ | 4.36^b^ | 1.89^bc^ | 6.44^b^ |
| **Verrucomicrobia** | 0.33^b^ | 1.23^b^ | 0.71^b^ | 1.62^b^ | 20.91^a^ | 4.94^b^ |
| **Bacteroidetes** | 5.72^a^ | 0.84^b^ | 1.03^b^ | 0.23^c^ | 0.08^c^ | 1.57^b^ |
| **Fusobacteria** | NA | 0.31 | 0.2 | 0.11 | 1.3 | 0.38 |
| **Spirochaetes** | NA | 0.05 | NA | NA | NA | 0.01 |
| **Tenericutes** | 0.02 | 0.05 | NA | 0.16 | 0.08 | 0.06 |
| **Chloroflexi** | 0.02 | NA | 0.04 | 0.04 | 0.07 | 0.03 |
| **TM7** | NA | NA | 0.01 | NA | NA | 0.00 |
| **SBR1093** | NA | NA | NA | 0.08 | NA | 0.02 |
| **Lentisphaerae** | NA | NA | NA | NA | 0.16 | 0.03 |
| **Others** | 0.04 | 0.41 | 0.25 | 0.39 | 1.61 | 0.54 |

**Supplementary Table 3.** The most abundant bacterial groups at genera level were detected in different habitats (gill, skin, stomach, hindgut and water). ANOVA multiple comparisons were used to test the significance among habitats.

|  | **Water (%)** | | **Gill (%)** | **Skin (%)** | **Stomach (%)** | **Hindgut (%)** | | **All samples (%)** |
| --- | --- | --- | --- | --- | --- | --- | --- | --- |
| ***Akkermansia*** | NA | | 0.33^b^ | 0.48^b^ | 1.26^b^ | 20.87^a^ | 4.59^b^ | |
| ***Pseudomonas*** | 0.96^e^ | | 23.54^c^ | 32.12^b^ | 63.98^a^ | 8.80^d^ | 25.88^b^ | |
| ***Synechococcus*** | 22.08^a^ | | 1.66^b^ | 0.08^c^ | 3.67^b^ | 1.74^b^ | 5.85^b^ | |
| ***Methylobacterium*** | NA | | 30.42^a^ | 26.11^a^ | 5.98^c^ | 1.38^c^ | 12.78^b^ | |
| ***Anaerofustis*** | NA | | NA | NA | NA | 1.36 | 0.27 | |
| ***Cetobacterium*** | NA | | 0.23^b^ | NA | 0.11^b^ | 1.30^a^ | 0.33^b^ | |
| ***Streptococcus*** | NA | | 5.5^a^ | 5.36^a^ | 2.29^a^ | 1.18^a^ | 2.87^a^ | |
| ***Arcobacter*** | 2.14^a^ | | 0.37^a^ | 0.36^a^ | NA | 0.84^a^ | 0.74^a^ | |
| ***Nocardia*** | NA | | NA | NA | 1.27 | 0.84 | 0.42 | |
| ***Ruminococcus*** | NA | | NA | NA | NA | 0.4 | 0.08 | |
| ***Desulfovibrio*** | NA | | NA | NA | NA | 0.36 | 0.07 | |
| ***SMB53*** | NA | | NA | NA | 0.12 | 0.23 | 0.07 | |
| ***Ralstonia*** | NA | | 13.18^a^ | 11.26^a^ | 0.87^c^ | 0.21^c^ | 5.10^b^ | |
| ***Desulfobulbus*** | NA | | NA | NA | NA | 0.1 | 0.02 | |
| ***Clostridium*** | NA | | NA | NA | NA | 0.09 | 0.02 | |
| ***Agrobacterium*** | NA | | 1.48^a^ | 1.17^a^ | 0.44^a^ | 0.07^a^ | 0.63^a^ | |
| ***Acinetobacter*** | NA | | 1.15^a^ | 1.06^a^ | 0.80^a^ | 0.05^a^ | 0.61^a^ | |
| ***Oxalobacter*** | NA | | NA | NA | NA | 0.05 | 0.01 | |
| ***Fusibacter*** | NA | | NA | NA | NA | 0.04 | 0.01 | |
| ***Mycobacterium*** | 0.41 | | NA | NA | 0.13 | 0.04 | 0.12 | |
| ***Photobacterium*** | NA | | 0.98^a^ | 1.06^a^ | 1.14^a^ | NA | 0.64 | |
| ***Roseibium*** | NA | | NA | NA | 0.36 | NA | 0.07 | |
| ***Shewanella*** | NA | | NA | NA | 0.27 | NA | 0.05 | |
| ***Paracoccus*** | NA | | 0.18 | NA | 0.26 | NA | 0.09 | |
| ***Rhodococcus*** | NA | | 0.79^a^ | 0.88^a^ | 0.24^a^ | NA | 0.38 ^a^ | |
| ***Sphingobium*** | NA | | 0.53^a^ | 0.48a | 0.14a | NA | 0.23 | |
| ***Vibrio*** | 0.41 | | 0.61^b^ | 1.05^a^ | 0.13^c^ | NA | 0.44 | |
| ***Marivita*** | 0.24 | | NA | NA | 0.12 | NA | 0.07 | |
| ***Polynucleobacter*** | NA | | 0.92 | NA | NA | NA | 0.18 | |
| ***Sphingomonas*** | NA | | 0.42 | 0.34 | NA | NA | 0.15 | |
| ***Kaistobacter*** | NA | | 0.37 | 0.48 | NA | NA | 0.17 | |
| ***Enterococcus*** | NA | | 0.27 | 0.65 | NA | NA | 0.18 | |
| ***Psychrobacter*** | 0.81 | | 0.26 | 1.02 | NA | NA | 0.42 | |
| ***Lactobacillus*** | NA | | NA | 0.65 | NA | NA | 0.13 | |
| ***Pseudoalteromonas*** | 1.8 | | NA | 0.4 | NA | NA | 0.44 | |
| ***Staphylococcus*** | NA | | NA | 0.35 | NA | NA | 0.07 | |
| ***Exiguobacterium*** | 1.08 | | NA | 0.31 | NA | NA | 0.28 | |
| ***ProteobacteriaCandidatus Portiera*** | | 3.19 | NA | NA | NA | NA | 0.64 | |
| ***Loktanella*** | 1.7 | | NA | NA | NA | NA | 0.34 | |
| ***Erythrobacter*** | 1.69 | | NA | NA | NA | NA | 0.34 | |
| ***Anaerospora*** | 1.15 | | NA | NA | NA | NA | 0.23 | |
| ***Candidatus Aquiluna*** | 1.15 | | NA | NA | NA | NA | 0.23 | |
| ***Flavobacterium*** | 1.1 | | NA | NA | NA | NA | 0.22 | |
| ***Thalassobacter*** | 0.43 | | NA | NA | NA | NA | 0.09 | |
| ***Idiomarina*** | 0.27 | | NA | NA | NA | NA | 0.05 | |
| ***Fluviicola*** | 0.27 | | NA | NA | NA | NA | 0.05 | |
| ***C39*** | 0.21 | | NA | NA | NA | NA | 0.04 | |
| ***Planococcus*** | 0.21 | | NA | NA | NA | NA | 0.04 | |
| ***Other*** | 58.72 | | 16.81 | 14.39 | 16.43 | 60.03 | 33.27 | |

**Supplementary Table 4.** The taxonomy of core OTU (the OTU shared by >80% samples and abundances>0.01) in five habitats (skin, gill, stomach, hindgut and water), and asterisks denote OTU that was significantly correlative with weight mass.

| **OUT ID** | **Phylum** | **Class** | | | | | | **Order** | **Family** | | | | **Genus** | | |  |  |  |  |
| --- | --- | --- | --- | --- | --- | --- | --- | --- | --- | --- | --- | --- | --- | --- | --- | --- | --- | --- | --- |
| **All samples** |  |  | |  | | | | |  | | | |  | | |  |  |  |  |
| **OTU_1** | Proteobacteria | Gammaproteobacteria | | Pseudomonadales | | | | | Pseudomonadaceae | | | | *Pseudomonas* | | |  |  |  |  |
| **OTU_2** | Proteobacteria | Alphaproteobacteria | | Rhizobiales | | | | | Methylobacteriaceae | | | | *Methylobacterium* | | |  |  |  |  |
| **OTU_4** | Firmicutes | Clostridia | | Clostridiales | | | | | Clostridiales_Incertae Sedis XIII | | | | *Anaerovorax* | | |  |  |  |  |
| **OTU_6** | Proteobacteria | Deltaproteobacteria | | Desulfovibrionales | | | | | Desulfovibrionaceae | | | | *-* | | |  |  |  |  |
| **OTU_788** | Proteobacteria | Deltaproteobacteria | | Desulfovibrionales | | | | | Desulfovibrionaceae | | | | *-* | | |  |  |  |  |
| **OTU_33295** | Firmicutes | Erysipelotrichia | | Erysipelotrichales | | | | | Erysipelotrichaceae | | | | *Holdemania* | | |  |  |  |  |
| **OTU_46724** | Firmicutes | Clostridia | | Clostridiales | | | | | Clostridiales_Incertae Sedis XIII | | | | *Anaerovorax* | | |  |  |  |  |
| **Gill** | | | | | | | | | | | | | | | | | | | |
| **OTU_1** | Proteobacteria | Gammaproteobacteria | | | | Pseudomonadales | | | | Pseudomonadaceae | | *Pseudomonas* | | | |  |  |  |  |
| **OTU_2** | Proteobacteria | Alphaproteobacteria | | | | Rhizobiales | | | | Methylobacteriaceae | | *Methylobacterium* | | | |  |  |  |  |
| **OTU_9** | Bacteroidetes | Sphingobacteriia | | | | Sphingobacteriales | | | | Chitinophagaceae | | *-* | | | |  |  |  |  |
| **OTU_13** | Proteobacteria | Alphaproteobacteria | | | | Rhizobiales | | | | Rhizobiaceae | | *Rhizobium* | | | |  |  |  |  |
| **OTU_23** | Chlamydiae | Chlamydiia | | | | Chlamydiales | | | | Parachlamydiaceae | | *Neochlamydia* | | | |  |  |  |  |
| **OTU_33295** | Firmicutes | Erysipelotrichia | | | | Erysipelotrichales | | | | Erysipelotrichaceae | | *Holdemania* | | | |  |  |  |  |
| **OTU_42368** | Proteobacteria | Betaproteobacteria | | | | Burkholderiales | | | | Burkholderiaceae | | *Ralstonia* | | | |  |  |  |  |
| **Skin** | | | | | | | | | | | | | | | | | | | |
| **OTU_1** | Proteobacteria | Gammaproteobacteria | | | | Pseudomonadales | | | | Pseudomonadaceae | | *Pseudomonas* | | | |  |  |  |  |
| **OTU_2** | Proteobacteria | Alphaproteobacteria | | | | Rhizobiales | | | | Methylobacteriaceae | | *Methylobacterium* | | | | |  |  |  |
| **OTU_9** | Bacteroidetes | Sphingobacteriia | | | | Sphingobacteriales | | | | Chitinophagaceae | | *-* | | | | |  |  |  |
| **OTU_42368** | Proteobacteria | Betaproteobacteria | | | | Burkholderiales | | | | Burkholderiaceae | | *Ralstonia* | | | | |  |  |  |
| **Table S4 continued** | | | | | | | | | | | | | | | | | | | |
| **Hindgut** | | | | | | | | | | | | | | | | | | | |
| **OTU_1** | Proteobacteria | | Gammaproteobacteria | | | Pseudomonadales | | | | Pseudomonadaceae | | | | *Pseudomonas* | | |  |  |  |
| **OTU_4** | Firmicutes | | Clostridia | | | Clostridiales | | | | Clostridiales_Incertae Sedis XIII | | | | *Anaerovorax* | | |  |  |  |
| **OTU_6** | Proteobacteria | | Deltaproteobacteria | | | Desulfovibrionales | | | | Desulfovibrionaceae | | | | *-* | | |  |  |  |
| **OTU_10** | Verrucomicrobia | | Verrucomicrobiae | | | Verrucomicrobiales | | | | Verrucomicrobiaceae | | | | *Akkermansia* | | |  |  |  |
| **OTU_11** | Verrucomicrobia | | Verrucomicrobiae | | | Verrucomicrobiales | | | | Verrucomicrobiaceae | | | | *Akkermansia* | | |  |  |  |
| **OTU_12** | Firmicutes | | Clostridia | | | Clostridiales | | | | Lachnospiraceae | | | | *Hespellia* | | |  |  |  |
| **OTU_14** | Firmicutes | | Clostridia | | | Clostridiales | | | | Ruminococcaceae | | | | *-* | | |  |  |  |
| **OTU_15** | Proteobacteria | | Deltaproteobacteria | | | Desulfovibrionales | | | | Desulfovibrionaceae | | | | *-* | | |  |  |  |
| **OTU_18** | Firmicutes | | Clostridia | | | Clostridiales | | | | Ruminococcaceae | | | | *-* | | |  |  |  |
| **OTU_25** | Proteobacteria | | Deltaproteobacteria | | | Desulfovibrionales | | | | Desulfovibrionaceae | | | | *-* | | |  |  |  |
| **OTU_44** | Verrucomicrobia | | Verrucomicrobiae | | | Verrucomicrobiales | | | | Verrucomicrobiaceae | | | | *Akkermansia* | | |  |  |  |
| **OTU_74** | Proteobacteria | | Deltaproteobacteria | | | Desulfovibrionales | | | | Desulfovibrionaceae | | | | *Desulfovibrio* | | |  |  |  |
| **OTU_788** | Proteobacteria | | Deltaproteobacteria | | | Desulfovibrionales | | | | Desulfovibrionaceae | | | | *-* | | |  |  |  |
| **OTU_1966** | Verrucomicrobia | | Verrucomicrobiae | | | Verrucomicrobiales | | | | Verrucomicrobiaceae | | | | *Akkermansia* | | |  |  |  |
| **OTU_33295** | Firmicutes | | Erysipelotrichia | | | Erysipelotrichales | | | | Erysipelotrichaceae | | | | *Holdemania* | | |  |  |  |
| **OTU_37134** | Proteobacteria | | Deltaproteobacteria | | | Desulfovibrionales | | | | Desulfovibrionaceae | | | | *-* | | |  |  |  |
| **OTU_43331** | Firmicutes | | Clostridia | | | Clostridiales | | | | Ruminococcaceae | | | | *-* | | |  |  |  |
| **OTU_45495** | Proteobacteria | | Deltaproteobacteria | | | Desulfobacterales | | | | Desulfobacteraceae | | | | *-* | | |  |  |  |
| **OTU_45499** | Firmicutes | | Clostridia | | | Clostridiales | | | | Lachnospiraceae | | | | *Dorea* | | |  |  |  |
| **OTU_46724** | Firmicutes | | Clostridia | | | Clostridiales | | | | Clostridiales_Incertae Sedis XIII | | | | *Anaerovorax* | | |  |  |  |
| **Stomach** | | | | | | | | | | | | | | | | | | | |
| **OTU_1** | Proteobacteria | Gammaproteobacteria | | | | | Pseudomonadales | | | Pseudomonadaceae | | | | *Pseudomonas* | | | |  |  |
| **OTU_2** | Proteobacteria | Alphaproteobacteria | | | | | Rhizobiales | | | Methylobacteriaceae | | | | *Methylobacterium* | | | |  |  |
| **OTU_4** | Firmicutes | Clostridia | | | | | Clostridiales | | | Clostridiales_Incertae Sedis XIII | | | | *Anaerovorax* | | | |  |  |
| **Table S4 continued** | | | | | | | | | | | | | | | | | | | |
| **OTU_6** | Proteobacteria | Deltaproteobacteria | | | | | Desulfovibrionales | | | Desulfovibrionaceae | | | | *Unclassified* | | | |  |  |
| **OTU_8** | Cyanobacteria | Cyanobacteria | | | | | Unclassified | | | Family II | | | | *GpIIa* | | | |  |  |
| **OTU_46724** | Firmicutes | Clostridia | | | | | Clostridiales | | | Clostridiales_Incertae Sedis XIII | | | | *Anaerovorax* | | | |  |  |
| **Water** | | | | | | | | | | | | | | | | | | | |
| **OTU_8** | Cyanobacteria | Cyanobacteria | | | - | | | | | | Family II | | | | *GpIIa* | | | |  |
| **OTU_28** | Actinobacteria | Actinobacteria | | | Actinomycetales | | | | | | Acidothermaceae | | | | *Acidothermus* | | | |  |
| **OTU_54** | Firmicutes | Bacilli | | | Bacillales | | | | | | Bacillales_Incertae Sedis XII | | | | *Exiguobacterium* | | | |  |
| **OTU_63** | Cyanobacteria | Cyanobacteria | | | - | | | | | | Family II | | | | *GpIIa* | | | |  |
| **OTU_68** | Proteobacteria | Alphaproteobacteria | | | Rhodobacterales | | | | | | Rhodobacteraceae | | | | *-* | | | |  |
| **OTU_72** | Actinobacteria | Actinobacteria | | | Acidimicrobiales | | | | | | Acidimicrobiaceae | | | | *Ilumatobacter* | | | |  |
| **OTU_78** | Bacteroidetes | Flavobacteriia | | | Flavobacteriales | | | | | | Flavobacteriaceae | | | | *Tenacibaculum* | | | |  |
| **OTU_92** | Proteobacteria | Gammaproteobacteria | | | Alteromonadales | | | | | | Pseudoalteromonadaceae | | | | *Pseudoalteromonas* | | | |  |
| **OTU_107** | Actinobacteria | Actinobacteria | | | Actinomycetales | | | | | | Microbacteriaceae | | | | *Agrococcus* | | | |  |
| **OTU_140** | Proteobacteria | Alphaproteobacteria | | | Sphingomonadales | | | | | | Erythrobacteraceae | | | | *Erythrobacter* | | | |  |
| **OTU_186** | Proteobacteria | Gammaproteobacteria | | | Gammaproteobacteria_incertae_sedis | | | | | | - | | | | *Congregibacter* | | | |  |
| **OTU_202** | Proteobacteria | Alphaproteobacteria | | | Kiloniellales | | | | | | Kiloniellaceae | | | | *Kiloniella* | | | |  |
| **OTU_7752** | Proteobacteria | Alphaproteobacteria | | | Rhodobacterales | | | | | | Rhodobacteraceae | | | | *-* | | | |  |
| **OTU_26261** | Cyanobacteria | Cyanobacteria | | | - | | | | | | Family II | | | | *GpIIa* | | | |  |
| **OTU_32877** | Cyanobacteria | Cyanobacteria | | | - | | | | | | Family II | | | | *GpIIa* | | | |  |
| **OTU_33198** | Proteobacteria | Alphaproteobacteria | | | Rhodobacterales | | | | | | Rhodobacteraceae | | | | *-* | | | |  |
| **OTU_39575** | Proteobacteria | Alphaproteobacteria | | | Rhodobacterales | | | | | | Rhodobacteraceae | | | | *Youngimonas* | | | |  |
| **OTU_41803** | Proteobacteria | Alphaproteobacteria | | | Rhodobacterales | | | | | | Rhodobacteraceae | | | | *-* | | | |  |
| **OTU_46944** | Cyanobacteria | Cyanobacteria | | | - | | | | | | Family II | | | | *GpIIa* | | | |  |
| **OTU_47076** | Proteobacteria | Alphaproteobacteria | | | Rhodobacterales | | | | | | Rhodobacteraceae | | | | *Nautella* | | | |  |
